# Daily Identity Dynamics in Adolescence Shaping Identity in Emerging Adulthood: An 11-Year Longitudinal Study on Continuity in Development

**DOI:** 10.1007/s10964-020-01370-3

**Published:** 2021-01-09

**Authors:** Andrik I. Becht, Stefanie A. Nelemans, Susan J. T. Branje, Wilma A. M. Vollebergh, Wim H. J. Meeus

**Affiliations:** 1 Research Center Adolescent Development, Utrecht University, Utrecht, the Netherlands; 2 Erasmus School of Social and Behavioural Sciences, Erasmus University Rotterdam, Rotterdam, the Netherlands; 3 Department of Interdisciplinary Social Sciences, Utrecht University, Utrecht, the Netherlands

**Keywords:** Daily identity dynamics, Adolescence, Emerging adulthood, Multi-level time-series, Within-person

## Abstract

According to identity theory, short-term day-to-day identity exploration and commitment processes are the building blocks for long-term development of stable commitments in emerging adulthood. This key assumption was tested in a longitudinal study including 494 individuals (43% girls, *M*_*age*_ T1 = 13.31 years, range 11.01–14.86 years) who were followed from adolescence into emerging adulthood, covering ages 13 to 24 years. In the first five years, adolescents reported on their daily identity processes (i.e., commitment, reconsideration and in-depth exploration) across 75 assessment days. Subsequently, they reported on their identity across four (bi-) annual waves in emerging adulthood. Findings confirmed the existence of a dual-cycle process model of identity formation and identity maintenance that operated at the within-person level across days during adolescence. Moreover, individual differences in these short-term identity processes in adolescence predicted individual differences in identity development in emerging adulthood. Specifically, those adolescents with low daily commitment levels, and high levels of identity reconsideration were more likely to maintain weak identity commitments and high identity uncertainty in emerging adulthood. Also, those adolescents characterized by stronger daily changes in identity commitments and continuing day-to-day identity uncertainty maintained the highest identity uncertainty in emerging adulthood. These results support the view of continuity in identity development from short-term daily identity dynamics in adolescence to long-term identity development in emerging adulthood.

## Introduction

Establishing a strong set of identity commitments is a crucial task both in adolescence and emerging adulthood (Erikson 1968; Schwartz et al. 2005). However, when adolescents transition to emerging adulthood, the development of firm commitments becomes increasingly important (Schwartz et al. 2005). For example, many emerging adults need to commit to life defining choices, such as commitments to a certain occupational career. Also, compared to the period of adolescence, continuing identity reconsideration, or identity uncertainty in emerging adulthood becomes increasingly related to ruminative exploration and depressive symptoms (Luyckx et al. 2013). Identity uncertainty in emerging adulthood thus represents an important risk factor for the development of mental health problems and psychopathology (Schulenberg et al. 2004). Therefore, it is vital to investigate why some emerging adults develop strong commitments whereas others continue to be uncertain about who they are. One possible important theoretical view to consider in this regard is that the development of long-term stable commitments is expected to emerge from short-term micro-level identity exploration and commitment processes in adolescence. Specifically, those adolescents with high commitment levels and low day-to-day fluctuations in their identity are expected to develop the strongest identity over time (Lichtwarck-Aschoff et al. 2008; Stephen et al. 1992). At the same time however, identity exploration and openness to change one’s identity commitments is also considered a vital aspect for positive identity development (Erikson 1968; Marcia 1966). Yet, an empirical test of these hypotheses is lacking. Moreover, whereas daily identity formation processes are considered highly personal and dynamic (Lichtwarck-Aschoff et al. 2008), surprisingly little is known about how these daily exploration and commitment processes actually affect each other at the within-person level. To answer these questions, the present study examined how adolescents’ identity processes affect each other across days and shape the development of stable commitments in emerging adulthood.

### Identity in Emerging Adulthood

In many Western countries the period in which individuals need to develop strong commitments is stretched beyond adolescence into the early twenties. Emerging adults need to make many life defining commitments such as a commitment to a certain study or occupation and certain interpersonal relationships. Theoretically, identity commitments are expected to progressively strengthen in emerging adulthood compared to adolescence (e.g., Waterman 1982). While it may be true that many emerging adults develop strong commitments, the strength of identity commitments as well as the amount of identity uncertainty or exploration continues to vary substantially between individuals (Schwartz et al. 2005).

While strong individual differences in identity development have been systematically shown in adolescence (e.g., Hatano and Sugimura 2017; and Meeus 2011 for a review of longitudina studies), so far only one longitudinal study has reported on different identity status trajectories in emerging adulthood (Luyckx et al. 2008). In this study, four identity status trajectories were identified, which closely resembled Marcia’s (1966) classical identity statuses: Individuals in identity *moratorium* have low commitments and high exploration of alternatives, *foreclosures* have relatively strong commitments and low exploration of alternative commitments, *achievers* displayed relatively high commitments, low exploration of alternatives and high in-depth exploration of current commitments, and a second class of *achievers* reported high commitments but high levels of exploration of alternative commitments as well. No identity diffusion status (characterized by low commitments and low exploration) was found in emerging adulthood (Luyckx et al. 2008). The absence of the identity diffusion status is not surprising since most emerging adults either already have explored identity alternatives and made commitments (e.g., commitments to a certain study or occupation) or are in the process of exploring and forming commitments. Further support for decreasing prevalence of identity diffusion comes from a meta-analysis demonstrating that the number of individuals in identity diffusion status is much lower in emerging adulthood compared to adolescence (Kroger et al. 2010). Similarly, a longitudinal interview study revealed only a small group of young adults (N = 7 out of 124 individuals) that continued to stay in a diffused identity status across ages 25–29 (Carlsson et al. 2016). Already in adolescence there is a systematic decrease in the number of adolescents in identity diffusion status (Meeus et al. 2010, 2012). In summary, different identity statuses have been found in adolescence. However, it remains unclear to what extent these identity statuses in adolescence correspondent with identity statuses in emerging adulthood. Based on limited longitudinal evidence in emerging adulthood, at least three identity statuses (i.e., moratorium, achievement and foreclosure), and no identity diffusion status can be expected.

### Daily Within-Person Identity Formation Processes in Adolescence

Yet, what factors might account for individual differences in identity status trajectories in emerging adulthood? Theoretically, short-term daily identity processes in adolescence might predict identity statuses in emerging adulthood (Lichtwarck-Aschoff et al. 2008). However, what are these short-term processes of identity development and how do they operate in adolescence? Recently developed identity models postulate two key processes of identity development in adolescence. These so-called dual-cycle models elaborate on Marcia’s (1966) identity status paradigm by not only concentrating on the process of identity formation but also on the process of evaluating and maintaining commitments (Luyckx et al. 2006; for reviews see Meeus 2011, 2018). Within these dual-cycle models, identity development is defined as a dynamic process. That is, in the *identity formation cycle* adolescents form commitments in a dynamic between considering identity alternatives (i.e., reconsideration) and making an identity choice (i.e., commitment). The *identity maintenance cycle* represents adolescents’ dynamic between commitment and active in-depth exploration of these commitments and serves the function of making these commitments more conscious and further strengthen them (Crocetti et al. 2008; Luyckx et al. 2006).

Identity is considered a dynamic self-organizing system that is shaped from day-to-day (Bosma and Kunnen 2001; Lichtwarck-Aschoff et al. 2008). Consistent with this dynamic view, identity formation and identity maintenance cycles are assumed to take place on a day-to-day basis as well. However, most studies, investigated identity development across long-term intervals, which is not informative on how identity developmental processes operate on a day-to-day basis (Lichtwarck-Aschoff et al. 2008). Therefore, the present study took a short-term approach in order to obtain a detailed perspective on how identity formation and maintenance cycles operate across adolescence.

Next to taking a detailed approach by studying identity from day-to-day, it has been argued that the process of identity development should be studied at the within-person level (Becht et al. 2017; Lichtwarck-Aschoff et al. 2008). That is, because group-based between-person effects are not necessarily the same, and can even be unrelated to the effects between variables at the within-person level (Hamaker et al. 2015; Molenaar and Campbell 2009). When taking a within-person approach to study identity formation one could test, for example, whether an increase in one’s commitment level relative to his or her own previous commitment level would relate to a decrease in identity reconsideration the next day, relative to his or her own previous level of reconsideration. In contrast, when studying this process at the group level, or between-person level one would examine the question whether a relatively higher score on commitment from one day to the next, compared to other adolescents would relate to a decrease in identity reconsideration, again, relative to other adolescents. As such, a within-person analytical approach provides a test whether these processes actually take place within the same persons across time, at the level were identity development is assumed to take place.

Unfortunately, most empirical studies examined identity formation and maintenance cycles at the between-person level and typically at longer time intervals. For instance, regarding the identity formation cycle, at the between-person level, adolescents’ higher commitment was negatively related to identity reconsideration, both cross-sectional (e.g., Crocetti et al. 2015; Luyckx et al. 2008), and longitudinal (i.e., 3-4 month interval between assessment waves; Pop et al. 2016). One between-person study tested short-term daily identity dynamics between commitment and reconsideration in early adolescence across days (Klimstra et al. 2010). This study used the same sample as the present study but only included the first 15 assessment days in early adolescence. They found that in both the interpersonal and educational identity domain, adolescents’ higher commitments on one day predicted less identity reconsideration the next day, as well as vice versa (Klimstra et al. 2010). In sum, these between-person findings tentatively support a daily identity formation cycle across shorter and longer time-intervals.

In addition, between-person studies also supported an identity maintenance cycle that operated in adolescence. For instance, higher commitments correlated with more in-depth exploration, both cross-sectional (Crocetti et al. 2015), and longitudinally across 3-6-month intervals (Luyckx et al. 2006; Pop et al. 2016), as well as across a 3-year interval (Meeus et al. 2002). These between-person finding suggest an adolescent identity maintenance cycle that operates across longer time intervals as well.

Only one study provided within-person evidence for a short-term identity formation cycle (van der Gaag et al. 2016). Specifically, first-year female college students were followed across 30 weeks (1 assessment each week). On average, when individuals increased in their level of commitments across days, they reported decreasing reconsideration of educational alternatives as well) (i.e., within-person correlations). In order to extend previous studies and obtain a more detailed picture of identity development, this study tested whether adolescents’ identity formation and maintenance cycles operated at the within-person level across days during adolescence.

### Daily Identity Formation in Adolescence and Long-Term Identity in Emerging Adulthood

A next question is whether and what aspects of short-term daily identity processes foster the development of a strong identity in emerging adulthood. Theoretically, youth with a high sense of sameness and continuity and low day-to-day fluctuations in their identity are expected to maintain strong and stable identity commitments over time adulthood (Erikson 1968; Lichtwarck-Aschoff et al. 2008). In order to capture the complex dynamic interplay of different identity processes within a system (i.e., an individual adolescent), research need to distinguish between different parameters at the daily level such as their (1) *level* of commitment and exploration processes, (2) the *stability* of these processes and, (3) *dynamic associations* between identity commitment and exploration processes (Lichtwarck-Aschoff et al. 2008).

Studies that focused on other aspects of the self, like self-esteem or self-concept clarity, have found that those individuals with more fluctuations (or instability) in their self-esteem across days reported lower levels of self-esteem as well as more adjustment problems (Campbell 1990; Kernis et al. 1989). Similarly, instability in daily identity reconsideration has been related to lower commitment levels three months later in early adolescence (Klimstra et al. 2010). Yet, the period of adolescence is also considered vital for identity exploration and the formation of new commitments. Yet, whether and individual differences in exploration-commitment dynamics predict later identity development remains unknown. Based on both the theoretical notion of sameness and continuity over time (Erikson 1968) as well as limited empirical evidence, it is expected that those adolescents with relatively high commitment levels that are stable across days (i.e., indicating a high sense of sameness and continuity across days), are likely to maintain strong commitments when they develop into emerging adulthood. In contrast, adolescents with low commitment levels, and lower stability in daily identity processes are expected to maintain high identity uncertainty when they grow older. In addition to the levels and stability of identity exploration and commitment dimensions, this study will explore how individual differences in daily exploration-commitment dynamics predict later identity.

## Current Study

The overarching aim of this longitudinal study was to test whether daily identity dynamics in adolescence could explain individual differences in long-term development of identity in emerging adulthood. To this end, the current study addressed three objectives. First, this study extends previous research by empirically testing whether different identity status trajectories exist in emerging adulthood (Objective 1). This study examined these trajectory statuses in two salient identity domains in emerging adulthood (i.e., the interpersonal and educational domains). In both identity domains at least three identity statuses were expected: (1) an *identity achievement* status (high commitments, high in-depth exploration, and very low reconsideration), (2) an *identity (fore)closure* status (moderate to high commitments, low in-depth exploration and low reconsideration), and (3) an *identity moratorium* status (low commitments, low in-depth exploration and high reconsideration).

Second, this study examined how identity dynamics operated on a daily basis at the within-person level from early to late adolescence (Objective 2). Based on dual-cycle identity process models (Crocetti et al. 2008; Luyckx et al. 2006), it is hypothesized for both the interpersonal and educational identity domains that (a) adolescents’ increasing commitments on one day predicted less identity reconsideration the next day, as well as vice versa (i.e., indicative of an identity formation cycle), (b) increasing commitment predicts increasing in-depth exploration the next day, as well as vice versa (i.e., supporting an identity maintenance cycle). Additionally, this study explored how reconsideration and in-depth exploration affected each other across days.

Third, this study tested whether individual differences in daily identity dynamics in adolescence predicted long-term identity trajectory statuses in emerging adulthood (Objective 3). Consistent with the notion of sameness and continuity (Erikson 1968), it is hypothesized that those adolescents with relatively high commitment levels that are relatively stable across days, were more likely to develop and maintain a strong identity in emerging adulthood as well.

## Methods

### Participants

Participants were 494 Dutch adolescents (43% girls, *M*_age_ T1 = 13.31, *SD* = 0.45) who joined the ongoing longitudinal project Research on Adolescent Development and Relationships Young cohort (RADAR-Y). For the current study, adolescents were followed into emerging adulthood until ages 24 years. Based on parent’s job level, the majority of the participants came from medium to high SES families (87.9%).

Missing values analyses on the daily identity reports indicated that each day, on average 68% of adolescents’ possible data points were completed. Because of the large number of data points relative to the number of participants, this study conducted Little (1988) on the daily identity measures per year. This MCAR test revealed a normed chi-square (χ^2^/df) ranging between 1.02 and 1.07 indicating that it is unlikely that study findings were biased as a result of missing values (Bollen 1989). Similarly, the MCAR test on the annual identity measures in emerging adulthood revealed a normed chi-square test of 1.21. Therefore, missing data were handled in M*plus* using Full Information Maximum Likelihood (FIML).

### Procedures

The current study used data from nine waves of RADAR-Y, with the same subjects being followed from 13 years until 24 years. Within the first five years the study adopted a measurement burst design in which adolescents participated in 3 measurement weeks in each of the five years. Thus, across the first five years there were 15 measurement weeks. Within each measurement week, participants filled out an online questionnaire tapping into their interpersonal and educational identity for 5 days in a row (i.e., from Monday through Friday), resulting in 5 days × 15 weeks = 75 daily assessments of identity. The initial measurement week (T1) took place in June, the second and third measurement weeks took place 3 and 6 months later, respectively. This same interval between measurement weeks was used across 5-years.

In addition to the daily diary assessments in the first five years, participants continued to complete identity questionnaires on both interpersonal and educational identity across four (bi) annual waves during emerging adulthood. These waves will be further referred to as T6 through T9. Measurement waves T5 and T6 were separated by a 1-year interval. Waves T6 and T7 were separated by a 1.5-year interval. T7 through T9 were separated by a two-year interval. Interpersonal identity was not assessed at T7 (i.e., three rather than four waves were available for interpersonal identity). Participants were recruited from central and western parts of the Netherlands (see for a detailed description of sample recruitment for example; Schwartz et al. 2011). All participants signed an informed consent form. The medical ethical committee of the University Medical Center Utrecht has approved the RADAR-study.

### Measures

#### Adolescent daily identity

Adolescents reported on their daily identity using the single-item version of the Utrecht-Management of Identity Commitments Scale (U-MICS; Klimstra et al. 2010). The item for identity commitment was “Today, I felt confident about myself because of my best friend/school” for the interpersonal and educational identity domain, respectively. For reconsideration the item reads: “Today, I felt that I could better look for a different best friend/school” (interpersonal and educational domain) and for in-depth exploration: Today, I often thought about my best friend/school” (interpersonal and educational domain). Items were rated on a 5-point Likert scale (1 = *completely untrue*, 5 = *completely true*). Reliability and validity of the single-item questions of the U-MICS has been supported (Becht, Branje et al. 2016; Becht, Nelemans et al. 2016; Klimstra et al. 2010). Moreover, longitudinal measurement invariance from early to late adolescence has been supported (Becht et al. 2016).

#### Emerging adult identity

Participants reported on their interpersonal and educational identity (bi-) annually in emerging adulthood using the full Utrecht-Management of Identity Commitments Scale (U-MICS; Crocetti et al. 2008; Meeus et al. 2010). The U-MICS includes 26 items that are rated on a response scale ranging from 1 (*completely untrue*) to 5 (*completely true*). Thirteen items tap into participants’ interpersonal identity, and 13 items tap into educational identity. Example items for commitment read: “My best friend/education gives me certainty in life” (interpersonal and educational commitment, respectively), “I often think it would be better to try to find a different best friend/education” (interpersonal and educational reconsideration), and “I think a lot about my best friend/education” (interpersonal and educational in-depth exploration, respectively). Reliability and factorial validity of the U-MICS has been widely supported across different samples, in different countries, and across boys and girls. Longitudinal measurement invariance has been established from adolescence into emerging adulthood (ages 10-25 years; e.g., Crocetti et al. 2015, 2008; Morsunbul et al. 2014). In the present study, Cronbach’s alphas for interpersonal identity ranged from 0.92 to 0.95 for commitment, from 0.92 to 0.93 for reconsideration, and from 0.77 to 0.86 for in-depth exploration, across waves. For educational identity, Cronbach’s alphas ranged from 0.93 to 0.95 for commitment, from 0.88 to 0.91 for reconsideration, and from 0.73 to 0.85 for in-depth exploration across waves.

Additional attrition analyses were conducted in order to examine whether young adults who dropped out over the course of the study differed on the study variables compared to those who did not drop out over the study period. This study examined differences on all W6 identity variables, including interpersonal and educational commitment, reconsideration and in-depth exploration. A multivariate analysis of variance (MANOVA) revealed no significant mean level differences on any of the study variables, *F*(6, 198) =1.73, *p* = 0.117, partial *η*^*2*^ = 0.05.

### Statistical Analyses

For the first aim, this study investigated whether individuals show different identity status trajectories in emerging adulthood. Specifically, this study tested the number and shape of developmental trajectories of commitment, in-depth exploration and reconsideration from age 18 to 24 years. To this end, the current study applied a multivariate Latent Class Growth Analyses (LCGA) for interpersonal identity and for educational identity separately. To decide upon the optimal number of latent classes, this study used the Sample Size Adjusted Bayesian Information Criterion (SSABIC) and the bootstrapped likelihood ratio test (BLRT; Nylund et al. 2007). A lower SSABIC value indicates a better fitting model and a significant BLRT indicates that a model with k classes fits better than a model with k – 1 classes. Furthermore, entropy, a standardized measure of qualification of individuals into latent trajectory classes, should be acceptable. Entropy values range between 0 and 1, with values of .75 or higher indicating good classification (Reinecke 2006). Every class needs to cover at least 10% of the sample for meaningful interpretation and subsequent analyses as small classes maybe too small to be meaningful or difficult to replicate (Muthén, and Muthén 2000). Finally, the content of the classes was evaluated. If an additional class was found to be a slight variation of a class solution with k-1 class, the most parsimonious class solution was favored.

For the second aim, this study tested how daily identity processes were related at the within-person level across adolescence. To this end, the current study applied Dynamic Structural Equation Modeling (DSEM; Asparouhov et al. 2017) in M*plus* 8.1 (Muthén and Muthén 1998–2017). DSEM is a multilevel extension of time series models that allows to test and describe daily autoregressive parameters (in the present case, stability of daily identity dimensions) as well as daily cross-lagged parameters, or dynamic effects, on intensive longitudinal data at the within-person level. In addition to modeling these within-person processes, individual differences in stability and cross-lagged parameters are estimated (Hamaker and Wichers 2017). To this end, the present study modeled within-person daily stability and cross-lagged effects between daily commitment, daily reconsideration, and daily in-depth exploration and allowed the estimated means, stability paths and cross-lagged effects to vary between-persons. In doing so, this study could test whether individual differences in mean level, stability and cross-lagged parameters predicted identity trajectory classes in emerging adulthood. This study used the available M*plus* TINTERVAL option to account for unequal time intervals between daily assessments (for more information see (Hamaker 2017; Muthen and Muthen 1998–2017). DSEM analyses were conducted for interpersonal and educational identity, separately.

For the third aim, this study combined the obtained results from objective 1 (establishing different identity status trajectories in emerging adulthood) and objective 2 (testing daily within-person identity dynamics in adolescence) to investigate whether individual differences in the daily level, stability and cross-lagged parameters in adolescence (ages 13–17 years) predicted long-term identity status trajectories in emerging adulthood (ages 18–24 years). To this end, the means of commitment, reconsideration and in-depth exploration across 75 days, the daily stability paths of these three dimensions, and the 6 possible cross-lagged effects between the identity dimensions were used as predictors of membership to the obtained identity status trajectories in emerging adulthood. The R3STEP command in M*plus* was employed for membership prediction, using logistic regression analyses (Asparouhov and Muthén 2014). Because a model including all predictors (i.e., means, stability paths and cross-lagged effects) of trajectory class membership was too complex, each predictor was tested separately. To account for multiple testing, a false discovery rate correction was applied (Benjamini et al. 2001).

## Results

### Objective 1: Establishing Long-Term Identity Status Trajectories in Emerging Adulthood

#### Long-term interpersonal identity trajectories

**Fig. 1:**
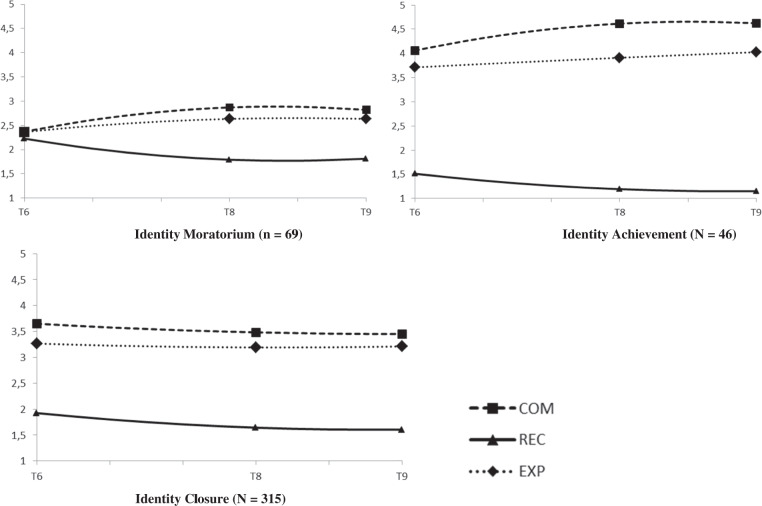
Estimated trajectories for interpersonal commitment, reconsideration, and exploration in-depth. COM interpersonal commitment, REC interpersonal reconsideration, EXP interpersonal in-depth exploration. Interpersonal identity was assesed at T6, T8, and T9. T6 and T8 were separated by a ≈ 3.5-year interval, T8 and T9 were separated by a 2-year interval

For interpersonal identity, a 3-class solution best fitted the data (Sample Size Adjusted Bayesian Information Criterion; SSABIC = 7192.05), BLRT < 0.001, Entropy 0.82. Although adding a 4th class further lowered the SSABIC (7068.18) and included a significant BLRT, *p* < 0.001, the fourth class closely resembled a class already captured with the 3-class solution. See online supplementary Fig. S1 where the estimated trajectories of the 4-class solution are also presented. This 4-class solution included a class too small (i.e., N = 28, 7% of the sample) for meaningful replication. Hence, for theoretical and parsimonious reasons the 3-class solution was maintained. See Table S1 for an overview of fit statistics of the 1-4 class solution. The estimated trajectories of the final 3-classs solution of commitment, reconsideration and in-depth exploration are presented in Fig. 1. The exact parameter estimates of the intercept and linear and quadratic slope factors of the latent classes can be found in the online supplementary material Table S2. Emerging adults in the first trajectory class (16%) showed relatively low commitment levels which increased over time, stable low levels of in-depth exploration and stable high reconsideration levels. This class was labeled the identity moratorium class. The second trajectory class (11%) showed the highest and increasing commitment levels, high and stable levels of in-depth exploration and very low decreasing identity reconsideration. Hence, this class was labeled the identity achievement class. The third trajectory class (73%) showed moderate and stable levels of commitment and in-depth exploration and low and decreasing identity reconsideration. Hence, this class was labeled the identity closure class.

#### Long-term educational identity trajectories

**Fig. 2:**
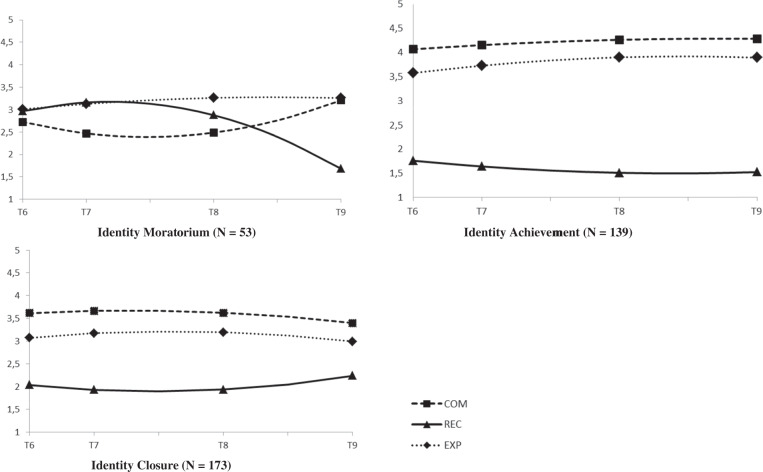
Estimated trajectories for educational commitment, reconsideration, and exploration in-depth. COM educational commitment, REC educational reconsideration, EXP educational in-depth exploration. Educational identity was assessed at T6, T7, T8, and T9. T6 and T7 were separated by a ≈1.5 year interval. T7 and T8, and T8 and T9 were separated by a 2-year interval

For educational identity, a 3-class solution showed the best fit to the data as well (SSABIC = 8048.16), BLRT < .001, entropy = 0.72). Although a slightly lower SSABIC suggested that a 4-class solution would better fit the data (SSABIC = 8007.25, and BLRT < 0.001) this additional 4th class closely reflected a class already captured with the 3-class solution (for more information, see supplementary Fig. S2 that shows the estimated trajectories of the 4-class solution). The 4-class solation included a class with only 32 participants (9% of the sample) and was therefore too small. Therefore, the 3-class solution was kept. See Table S4 for all fit statistics of the 1-4 class solution. The estimated trajectories of commitment, in-depth exploration, and reconsideration of the 3-class solution are presented in Fig. 2. The parameter estimates of the intercept and slope factors of the latent classes can be found in the online supplementary material Table S2. Emerging adults in the first trajectory class (15%) showed the lowest and decreasing identity commitments, which increased again over time, stable low in-depth exploration, and the highest and stable reconsideration levels which decreased over time. Hence, this class was labeled the identity moratorium class. The second trajectory class (39%) showed the highest baseline levels of commitment and in-depth exploration. Reconsideration was very low across waves. Hence, this class was labeled the identity achievement class. The third trajectory class (46%) showed relatively high and stable identity commitments over time, relatively high and stable levels of in-depth exploration, which slightly decreased around T9. Reconsideration was relatively low but increased somewhat over time. This class was labeled the identity closure class.

The number and shape of the developmental trajectory statuses were fairly similar across the interpersonal and educational identity domain. Despite the known limitations when using class assignments in subsequent separate analyses (Vermunt 2010), this study tested a 3 × 3 cross-tabulation on both the interpersonal (3-classes) and educational (3 classes) membership variables. This analysis revealed significant overlap in the distribution of participants across the both identity domains, χ^2^ (4) = 18.08, *p* = 0.001, *φ*_c_ = 0.16. Yet, there were also differences. For example, almost half of the emerging adults in interpersonal moratorium status were in educational closure status. Moreover, around 40% of individuals in interpersonal closure status were in educational identity achievement status. These findings emphasize the importance to differentiate between identity domains (Goossens 2001).

### Objective 2: Within-Person Daily Identity Dynamics Across Adolescence

**Fig. 3:**
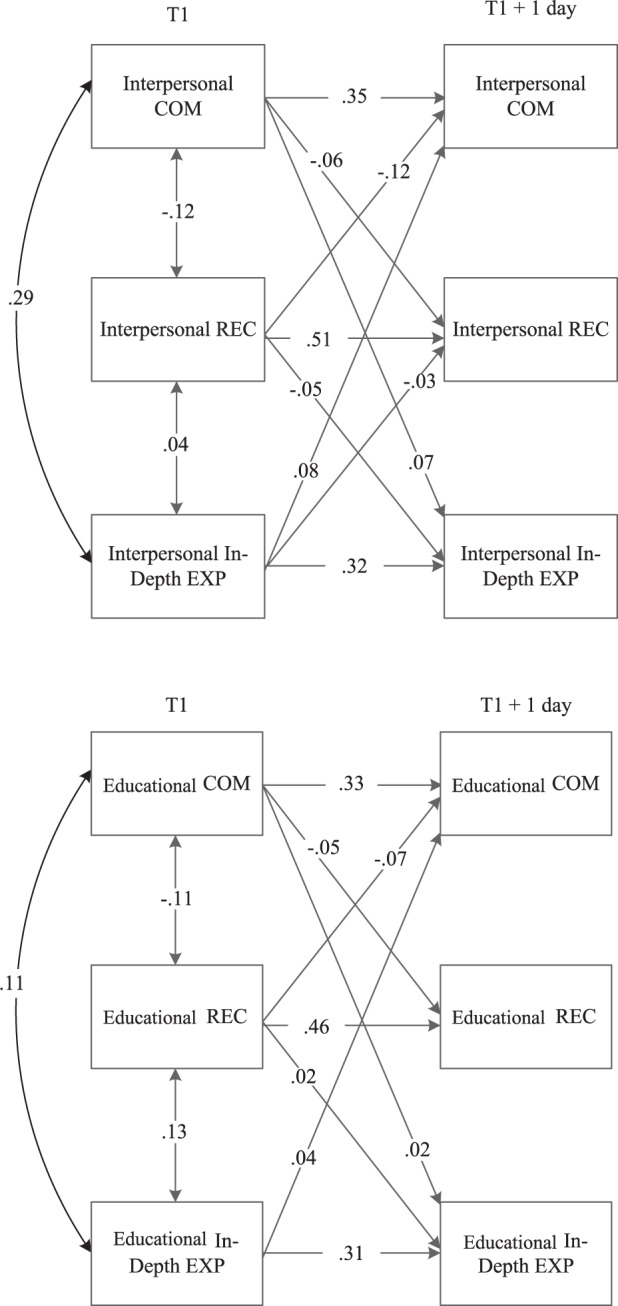
Significant standardized results of daily within-person cross-lagged models for (top) interpersonal identity formation processes and (bottom) educational identity formation processes. COM commitment, REC reconsideration, EXP in-depth exploration. Arrows displayed were significant at *p* < 0.05

Next, this study tested how identity formation processes operated at the daily level within-persons, using a DSEM approach. The analytical model is presented in supplementary material Fig. S3. Standardized within-person concurrent associations, stability paths, and cross-lagged effects are presented in Fig. 3.

#### Interpersonal identity dynamics

Based on all daily measurement days, the concurrent association between interpersonal commitment and reconsideration was negative, commitment and in-depth exploration were positively associated, and reconsideration and in-depth exploration were positively related. Concerning daily stability paths, daily reconsideration showed the highest stability, followed by commitment and in-depth exploration. Daily stability of identity reconsideration was significantly higher (*p* < 0.05) compared to the stability of commitment and in-depth exploration, which did not differ significantly from each other. Concerning the daily within-person cross-lagged effects, results supported a daily identity formation cycle. Specifically, when adolescents showed higher commitment levels on one day, they reported lower identity reconsideration the next day. And vice versa, increasing reconsideration predicted decreasing commitment one day later. However, the effect of reconsideration predicting less identity commitment the next day was significantly stronger than the other way around. Also, supporting the hypothesized identity maintenance cycle, a within-person increase in commitment predicted more in-depth exploration the next day, as well as the other way around. Additionally, exploration of the daily dynamic between reconsideration and in-depth exploration revealed that more identity reconsideration predicted less in-depth exploration the next day and vice versa.

#### Educational identity dynamics

Results revealed a negative concurrent association between educational commitment and reconsideration, and positive associations between commitment and in-depth exploration as well as between reconsideration and in-depth exploration. Similar to findings for interpersonal identity, daily stability of reconsideration was significantly higher compared to the stability of commitment and in-depth exploration. Stability of commitment and in-depth exploration did not differ significantly in strength. Regarding daily within-person cross-lagged effects, study results again supported a daily identity formation cycle; When adolescents’ commitments increased, they reported less identity reconsideration the next day as well as vice versa. Also, supporting a daily identity maintenance cycle, a within-person increase in commitment level predicted more in-depth exploration the next day, and vice versa. The present exploratory study aim to test the daily dynamic between reconsideration and in-depth exploration revealed that increasing reconsideration predicted more in-depth exploration the next day but not vice versa.

### Objective 3: Daily Identity Processes in Adolescence Predicting Long-Term Identity Profiles in Emerging Adulthood

#### Interpersonal identity

Table 1 shows the exact parameter estimates for daily interpersonal identity dynamics predicting identity status trajectories in emerging adulthood. First, the identity achievement class served as the reference class to determine how emerging adults in identity moratorium differed from emerging adults in the achievement class. These analyses revealed that when adolescents reported higher commitment levels and more in-depth exploration across days, they were less likely to follow the identity moratorium status trajectory compared to the achievement status trajectory in emerging adulthood (odds ratio; OR = 0.31 and 0.44 for commitment level and in-depth exploration, respectively). Yet, when adolescents reported higher reconsideration levels, they were more likely to follow the identity moratorium trajectory in emerging adulthood (OR = 2.23). In addition to higher levels of identity commitment, more in-depth exploration and lower reconsideration levels, emerging adults in identity achievement status showed a less strong negative daily dynamic between commitment and reconsideration the next day compared to the identity moratorium status trajectory. Thus, when an increase in commitment was less strongly related to a sudden drop in identity reconsideration the next day, emerging adults were less likely to follow the identity moratorium trajectory and more likely to follow the identity achievement status trajectory (OR = 0.54).

**Table 1:** Raw parameter estimates and odds ratios for daily interpersonal identity dynamics across T1–T5 predicting class membership of developmental trajectories of annual identity across T6–T9

Class Membership T6–T9									
	Identity moratorium vs achievement			Identity moratorium vs identity closure			Identity closure vs achievement		
Model	*Estimate*	*SE*	*OR*	*Estimate*	*SE*	*OR*	*Estimate*	*SE*	*OR*
Daily identity (T1–T5)									
COM level	−1.18	0.28	0.31***	−0.88	0.16	0.41***	−0.30	0.27	0.74
REC level	0.80	0.24	2.23**	0.42	0.16	1.52*	0.38	0.21	1.46
EXP level	−0.83	0.25	0.44**	−0.25	0.15	0.78	−0.58	0.23	0.56*
STAB COM	0.02	0.23	1.02	−0.02	0.17	0.98	0.04	0.19	1.04
STAB REC	0.38	0.23	1.46	0.15	0.14	1.16	0.23	0.22	1.26
STAB. EXP	0.06	0.21	1.06	0.32	0.16	1.38	−0.26	0.19	0.77
REC→ COM	0.23	0.22	1.26	0.15	0.17	1.16	0.08	0.19	1.08
COM→REC	−0.61	0.21	0.54**	−0.45	0.16	0.64**	-0.16	0.17	0.85
EXP→COM	−0.02	0.24	0.98	0.46	0.16	1.58**	−0.48	0.23	0.62
COM→EXP	−0.20	0.22	0.82	−0.20	0.14	0.82	−0.00	0.22	1.00
EXP→REC	−0.32	0.25	0.73	−0.13	0.16	0.88	−0.23	0.20	0.79
REC→EXP	−0.23	0.20	0.79	0.13	0.16	1.14	−0.36	0.23	0.70

*COM level* commitment level across days, *REC level* reconsideration level across days, *STAB COM* stability daily commitment, *STAB REC* stability daily reconsideration, *REC→COM* reconsideration predicting commitment the next day, *COM→REC* commitment predicting reconsideration the next day, *STAB. EXP* = stability of in-depth exploration, *EXP→COM* in-depth exploration predicting commitment the next day, *COM→EXP* commitment predicting in-depth exploration the next day, *EXP→REC* in-depth exploration predicting REC the next day, *REC→EXP* reconsideration predicting in-depth exploration the next day

*p < 0 .05; **p < 0.01; ***p < 0 .001

Please note that this interpretation of a negative predictor (in this case the raw parameter estimate of the cross-lagged effect between commitment and reconsideration predicting class membership is −0.61, with OR = 0.54, see Table 1) with negative means can be difficult to conceptualize. Therefore, please find a brief guideline on the interpretation of these effects here. Concerning the previous described predictor, it was found that the daily lagged effect between commitment and reconsideration, negatively predicted class membership to the identity moratorium status (i.e., −0.61, see Table 1). However, the average within-person cross-lagged effect between commitment and reconsideration is also negative (i.e., COM → REC = −0.06, see Fig. 3). Therefore, the effect of this daily cross-lagged effect predicting membership to the moratorium identity trajectory class implies that when this lagged effect goes up (i.e., the lagged effect of commitment on one day becomes more positively related to reconsideration the next day), the likelihood of being in identity moratorium versus achievement goes down.

Next, emerging adults in identity moratorium were compared with identity closures (reference class). Again, adolescents with higher commitment levels were less likely to follow the identity moratorium trajectory compared to the closure trajectory (OR = 0.41). Whereas higher reconsideration levels in adolescence predicted an increasing likelihood to follow the identity moratorium status trajectory in emerging adulthood, relative to identity closure status (OR = 1.52). With regard to the daily identity dynamics, emerging adults were again less likely to follow the identity moratorium trajectory when increasing commitments on one day was less strongly related to a steep drop in identity reconsideration the next day when they were adolescents (OR = 0.64). Also, when an adolescent’s increase of in-depth exploration was followed by a stronger increase in commitment the next day, they were more likely to follow the identity moratorium trajectory in emerging adulthood relative to the identity closure status (OR = 1.58). Or stated the other way around, those emerging adults in identity closure status already showed a less strong daily dynamic between in-depth exploration and commitment when they were adolescents.

Finally, emerging adults in identity closure status were compared with emerging adults in identity achievement status (reference class). Individuals who showed higher levels of in-depth exploration in adolescence were less likely to follow the emerging adulthood closure trajectory status (OR = 0.56) and more likely to follow the achievement trajectory status. Daily stability and cross-lagged effects in adolescence did not further differentiate identity closures from identity achievers in emerging adulthood. In conclusion, findings indicated that especially daily identity levels in adolescence were the most consistent predictors of identity status trajectories in emerging adulthood. A limited number of daily dynamic effects in adolescence predicted identity status trajectories in emerging adulthood.

#### Educational identity

Table 2 shows the exact parameter estimates for daily educational identity dynamics predicting long-term identity status trajectories in emerging adulthood. First, the identity moratorium class was compared to the achievement reference class. Results indicated that when adolescents reported higher commitment levels, they were less likely to follow the identity moratorium trajectory in emerging adulthood compared to the identity achievement trajectory class (OR = 0.41). However, when reconsideration levels as well as the daily stability of reconsideration were higher in adolescence, participants were more likely to follow the identity moratorium trajectory in emerging adulthood (OR = 2.59 and OR = 2.34, for reconsideration levels and stability, respectively). Finally, when an increase in identity reconsideration was followed by a steeper increase of active in-depth exploration, those adolescents were more likely to follow the identity moratorium trajectory in emerging adulthood (OR = 2.05).

**Table 2:** Raw parameter estimates and odds ratios for daily educational identity dynamics across T1–T5 predicting class membership of developmental trajectories of annual identity across T6–T9

Class membership T6–T9									
	Identity moratorium vs achievement			Identity moratorium vs identity closure			Identity closure vs achievement		
Model	*Estimate*	*SE*	*OR*	*Estimate*	*SE*	*OR*	*Estimate*	*SE*	*OR*
Daily identity (T1–T5)									
COM Level	−0.90	0.23	0.41***	−0.52	0.20	0.59	−0.38	0.19	0.68
REC Level	0.95	0.19	2.59***	0.66	0.19	1.93***	0.29	0.18	1.34
EXP Level	−0.27	0.17	0.76	0.12	0.17	1.13	−0.39	0.17	0.78
STAB COM	0.42	0.18	1.52	0.30	0.17	1.35	0.12	0.17	1.13
STAB REC	0.85	0.19	2.34***	0.72	0.19	2.05***	0.13	0.16	1.14
STAB. EXP	−0.20	0.19	0.82	−0.04	0.19	0.96	−0.16	0.16	0.85
REC→ COM	−0.20	0.17	0.82	−0.43	0.19	0.65	0.23	0.18	1.26
COM→REC	−0.35	0.21	0.70	−0.23	0.21	0.79	−0.12	0.17	0.89
EXP→COM	0.23	0.18	1.26	0.38	0.18	1.46	−0.15	0.16	0.86
COM→EXP	0.15	0.18	1.16	0.20	0.19	1.22	−0.05	0.16	0.95
EXP→REC	0.38	0.19	1.46	0.29	0.20	1.34	0.09	0.16	1.09
REC→EXP	0.72	0.20	2.05***	0.46	0.19	1.58	0.26	0.18	1.30

*COM level* commitment level across days, *REC level* reconsideration level across days, *STAB COM* stability daily commitment, *STAB RE*C stability daily reconsideration, *REC→COM* reconsideration predicting commitment the next day, *COM→REC* commitment predicting reconsideration the next day, STAB. EXP stability of in-depth exploration, *EXP→COM* in-depth exploration predicting commitment the next day, *COM→EXP* commitment predicting in-depth exploration the next day, *EXP→REC* in-depth exploration predicting REC the next day, *REC→EXP* reconsideration predicting in-depth exploration the next day

*p < 0.05; **p < 0.01; ***p < 0.001

Next, emerging adults in identity moratorium were compared with identity closures (reference class). Again, those adolescents with higher levels of daily identity reconsideration as well as higher daily stability of reconsideration were more likely to follow the identity moratorium trajectory class relative to the identity closure trajectory class (OR = 1.93 and 2.05 for reconsideration level and stability, respectively). No other significant differences in daily adolescent identity dynamics between the identity closure and moratorium status were found.

No other significant differences in daily stability and dynamics were found when comparing emerging adults in identity closure status with emerging adults in identity achievement status. Note, however, that higher levels of in-depth exploration predicted membership to the identity achievement trajectory status compared to closures status. However, this effect was not significant anymore after applying the false discovery rate (FDR) correction to account for multiple testing. In sum, individual differences in daily educational identity levels during adolescence were the most consistent predictors of educational identity trajectory statuses in emerging adulthood. This study found some evidence for daily dynamic effects predicting later identity status trajectories in emerging adulthood but overall these linkages were less prevalent compared to daily identity levels.

## Discussion

Does the development of stable identity commitments in emerging adulthood emerge from short-term micro-level identity exploration and commitment processes in adolescence? While a large part of longitudinal studies has documented identity maturation across adolescence, little is known about how daily identity formation processes take place within-persons, and the type of daily identity formation processes that predict long-term identity development in emerging adulthood. The present study addressed these gaps in the literature. This study first established different identity status trajectories in emerging adulthood and found no identity diffusion status. Second, this study obtained consistent support for a within-person daily dual-cycle process of identity formation and maintenance across identity development in adolescence. Third, pertaining to the main aim, short-term daily identity levels in adolescence predicted identity statuses in emerging adulthood. Moreover, a limited number of short-term dynamic effects that reflect openness to identity change in adolescence predicted identity statuses in emerging adulthood.

### Identity in Emerging Adulthood

As predicted, in both the interpersonal and educational identity domain three identity status trajectories in emerging adulthood were identified: Moratorium, achievement and closure identity status trajectories. Emerging adults in identity *moratorium* showed a classical profile of weak identity commitments, no active processing of current commitments (low in-depth exploration) and the highest levels of considering alternative commitments over time. Emerging adults in the identity *achievement* trajectory class maintained the highest commitments, high in-depth exploration and showed the lowest and decreasing levels of identity reconsideration over time. Finally, identity *closures* showed in-between strength of identity commitments, less active thinking about these commitments compared to the identity achievement status (i.e., relatively lower in-depth exploration) and low reconsideration of alternative commitments over time.

Three findings stand out from the current longitudinal investigation of identity status trajectories in emerging adulthood. First, emerging adults showed stable individual differences in the extent to which they developed a clear sense of identity. One of these stable individual differences was the continuing existence of a subgroup of emerging adults in identity moratorium. This subgroup of individuals keeps struggling with identity issues, beyond adolescence into emerging adulthood, which also puts them at risk for the development of psychosocial adjustment problems (Luyckx et al. 2008). Second, this study did not find emerging adults in identity diffusion, an identity status that has been consistently found in adolescence. This contrasting finding between the period of adolescence and emerging adulthood further corroborates the notion of continuing identity maturation in emerging adulthood (Meeus 2011) and is consistent with previous longitudinal studies in adolescence that showed a significant decrease in the prevalence of identity diffusion status during adolescence (Meeus et al. 2012, 2010). Previous work that applied a similar dual-cycle process model to study identity development during emerging did not find an identity diffusion trajectory status either (Luyckx et al. 2008). However, based on longitudinal interview data, prior work identified a very small group (N = 7 out of 124 participants) of young adults still in identity diffusion status at ages 25 and 29 (Carlsson et al. 2016). Together, these findings highlight that at some point during the development into emerging adults, the large majority of individuals either have made certain commitments, or at least considered some identity commitments. Third, findings further support a dual-cycle perspective across development from adolescence into emerging adulthood. That is, a large majority (> 80%) of emerging adults were in identity closure or achievement status, whereas around 50% of adolescents have been found to follow an identity moratorium status trajectory (Becht, Nelemans, et al. 2016). These findings suggest that over time, individuals become less involved in the process of identity formation and continuing identity reconsideration but move into the process of either active (i.e., identity achievers, who actively explore their commitments in-depth) or passive (i.e., identity closures, who do not actively explore their commitments in-depth) maintenance of their identity commitments (Meeus 2018).

### Daily Within-Person Identity Processes in Adolescence

Consistent with a dual-cycle process model of identity development, this study supports the existence of both an identity formation and identity maintenance cycle in adolescence that operated on a daily basis at the within-person level. First, concerning the identity formation cycle, results confirm and extend previous findings that when adolescents made a commitment on one day, they reconsidered their identity less the next day, as well as vice versa (Klimstra et al. 2010; van der Gaag et al. 2016). These dynamics operated in both the interpersonal and educational identity domain. Second, consistent with theory and prior work on between-person annual associations between commitment and in-depth exploration (Meeus et al. 2002; Pop et al. 2016), this study found support for a daily identity maintenance cycle that operates at the within-person level: Those adolescents that increased their commitments from one day to the next were more likely to actively explore these commitments in-depth one day later. And, vice versa, when adolescents showed an increase of in-depth exploration, they reported stronger commitments the next day as well. This identity maintenance cycle operated both in the interpersonal and educational identity domains.

Next to the hypothesized and confirmed identity formation and maintenance cycles, this study explored whether identity reconsideration and in-depth exploration affected each other on a day-to-day basis. Within the interpersonal identity domain, increasing reconsideration predicted a *decrease* of in-depth exploration the next day, and vice versa. In contrast, within the educational identity domain, increasing reconsideration on one day predicted a slight *increase* (rather than decrease) of in-depth exploration the next day, but not vice versa. These contrasting findings across different identity domains tentatively support the idea of open versus closed identity domains in which developmental patterns can differ (Becht, Nelemans et al. 2016; Meeus et al. 2002). Especially during adolescence, interpersonal relationships are more open to change (Branje et al. 2007). Therefore, within this more open to change interpersonal identity domain, a negative dynamic between reconsideration and in-depth exploration might facilitate the process of identity change. That is, an increase in uncertainty about one’s interpersonal commitments further lowers the process of maintaining these commitments as reflected in decreasing in-depth exploration the next day. As such, this negative dynamic further weakens present commitments and makes way for changing one’s interpersonal commitments.

In contrast, there was no positive dynamic between reconsideration and in-depth exploration in the more closed educational identity domain. When adolescents start having doubts about their commitments to school, they showed an increase in active reflection and in-depth exploration upon these commitments the next day. This might result from adolescents’ awareness that moving to another school is not very likely. As a result, adolescents may start reflecting actively upon their commitment to school, possibly to try to find something positive about their commitment to school in order to maintain their school commitments. Note however, that the associations between reconsideration and in-depth exploration were among the weakest. Therefore, future studies are needed to replicate this finding across different identity domains. Although most daily lagged effects were modest in size, these effects are still meaningful, given the relatively high stability of identity processes across days. High stability can dramatically attenuate lagged effects. Therefore, small lagged effects are considered more meaningful when the stability effects are larger (Adachi and Willoughby 2015), as was the case in the present study.

Taken together, findings are consistent with the view that adolescents come to develop and maintain their identity commitments in a continuing day-to-day dynamic (Lichtwarck-Aschoff et al. 2008). Within these daily dynamics adolescents choose commitments and employ different ways of exploring alternative commitments and reflect on their present commitments to further strengthen their identity over time (Crocetti et al. 2008; Luyckx et al. 2006). These dynamics operated at the within-person level. That is, this study tested whether change relative to one’s own previous commitments impact subsequent reconsideration and in-depth exploration processes for that individual, and vice versa. These within-person associations converge with previously reported between-person associations between these identity processes (e.g., Klimstra et al. 2010; Pop et al. 2016). This convergence illustrates, for example, that both higher reconsideration levels relative to other individuals as well as compared to their own previous reconsideration levels is a risk factor for subsequent weakening of identity commitments both compared to others as well as well as compared to their own previous commitment levels. However, this within-person approach to study identity is most consistent with Marcia’s (1966, 1993) identity status interview, which is also focused on the assessment of within-person processes. For example, in the identity status interview, adolescents are asked when they made certain commitments, as well as whether they changed in their exploration of (alternative) identity choices over time. Adolescents might also differ from each other in how these identity processes are associated at the within-person level.

### Daily Identity Formation in Adolescence and Long-Term Identity in Emerging Adulthood

Therefore, the third aim was to test whether individual differences in day-to-day identity exploration and commitment processes predicted why some of these adolescents develop into emerging adults with a strong identity whereas others remain uncertain about who they are. The current study investigated different parameters in adolescence to capture daily identity development over time. Specifically, for each individual this study estimated their level and stability of daily identity processes as well as daily dynamic effects between those processes. Overall, longitudinal findings support the view that those adolescents with relatively strong and stable commitments across days in adolescence, were most likely to develop and maintain the strongest identity in emerging adulthood (Lichtwarck-Aschoff et al. 2008).

#### Interpersonal identity domain

With respect to the level on identity dimensions, those adolescents with high daily levels of interpersonal identity commitment, high levels of in-depth exploration and low levels of identity reconsideration were most likely to be in the high commitment identity achievement and closure statuses in emerging adulthood. In contrast, adolescents with low commitment levels, low in-depth exploration and high levels of reconsideration were most likely to follow the identity moratorium status in emerging adulthood.

Next to their lower levels of identity commitment and in-depth exploration and higher reconsideration levels, emerging adults in interpersonal identity moratorium showed a stronger negative day-to-day association between commitment and reconsideration in adolescence as well (compared to the identity closure and achievement status). Thus, when adolescents made a commitment on one day that was followed by a steeper temporal drop in reconsidering identity alternatives, they were more likely to be in identity moratorium in emerging adulthood, relative to the closure and achievement identity status. As such, these adolescents were characterized by relatively higher instability, or fluctuations during the process of identity formation across days. Combined with their lower levels of commitments and already higher levels of identity reconsideration, these findings suggest that those adolescents might temporarily stop with reconsidering identity alternatives following a commitment in order to be temporarily freed from continuing identity uncertainty. On a short-time scale their daily dynamic between commitment making and reconsideration might represent a continuing Moratorium-Achievement-Moratorium-Achievement cycle (MAMA-cycle; Stephen et al. 1992). Over time, however, this MAMA-cycle did not result in a stable sense of identity, since these adolescents continued to struggle with identity issues and stayed in identity moratorium status in emerging adulthood. In addition, study results support the view that individuals in interpersonal identity (fore)closure status do not show a history of active identity exploration before making firm commitments (Marcia 1966): Those emerging adults in the interpersonal identity closure status showed a weaker daily-dynamic between in-depth exploration and commitment when they were adolescents compared to individuals in identity moratorium status (i.e., the EXP→COM dynamic in Table 1). The stability of identity processes as well as dynamic effects between reconsideration and in-depth exploration did not predict later identity. Overall, daily levels of interpersonal identity processes were the most consistent predictors of later identity status trajectories.

#### Educational identity domain

Similar to the interpersonal identity domain, those adolescents with lower commitment levels and higher reconsideration levels were more likely to follow the identity moratorium status trajectory in emerging adulthood compared to both the closure or achievement status trajectory. In addition to these differences in daily levels, adolescents with higher stability in their day-to-day reconsideration of identity alternatives were at risk of following an identity moratorium status trajectory in emerging adulthood, both compared to identity achievers and closures. Thus, this finding was replicated across different reference groups. A higher day-to-day stability (or autoregressive) parameter has been interpreted as representing one’s individual inertia, which indicates the degree to which a person’s state is resistant to change (Kuppens et al. 2010). As such, this finding suggests that those adolescents with a higher daily stability of reconsideration might be stuck in a process of continuing identity exploration, or identity uncertainty, which undermines the formation of strong commitments over time.

Finally, this study found that when adolescents’ increasing educational reconsideration predicted a stronger increase of in-depth exploration the next day, they were at risk for following the identity moratorium trajectory in emerging adulthood compared to the identity achievement trajectory. This finding suggests that those adolescents with a more vibrant daily dynamic of continuing identity uncertainty and reflection upon their commitments are at risk of staying in an identity status in emerging adulthood that is characterized by high identity uncertainty as well. Similar to the interpersonal identity domain, the most important predictors of identity status trajectories in emerging adulthood were the daily identity levels in adolescence. In addition, two dynamic effects, reflecting higher daily identity uncertainty predicted continuing identity uncertainty in emerging adulthood as well.

### Implications for Theory and Developmental Outcomes

A key assumption of identity theory in particular and developmental science in general is that short-term processes are the driving force behind long-term development (see Bosma and Kunnen 2001; Lichtwarck-Aschoff et al. 2008 for a discussion on identity theory specifically, and Meeus 2016; Nesselroade and Molenaar 2010 on adolescent development in general). Study findings on the linkages between short-term identity processes and long-term identity development in emerging adulthood support this assumption. Especially adolescents’ daily levels predicted their later identity in emerging adulthood. Those adolescents with low daily commitment levels as well as continuing short-term identity fluctuations were more likely to carry this identity uncertainty into the next developmental phase of emerging adulthood. Besides, this study revealed the first empirical evidence that those adolescents who were more flexible in their daily identity formation processes (evidenced by *less* strong negative commitment-reconsideration dynamics) developed the strongest identity in emerging adulthood. By focussing on daily identity dynamics, this study was able to test and confirm that openness to identity change in adolescence is an important long-term predictor of identity achievement in emerging adulthood. As such, these findings indicate continuity in identity development from short-term processes in adolescence to long-term development of identity in emerging adulthood.

This study provided new insight into the type of identity formation processes that predict maladaptive long-term identity development and which identity formation processes predict the development of a strong and stable identity in emerging adulthood. These daily identity formation processes may be an important predictor of other developmental outcomes in adolescence and emerging adulthood as well, including externalizing and internalizing behaviors (for reviews see Meeus 2011, 2018; Van Doeselaar et al. 2018). For instance, recent work revealed that increasing identity reconsideration in adolescence predicted increasing depressive symptoms one year later but not vice versa. This unidirectional effect took place at the within-person level and was replicated across two large adolescent samples (Becht et al. 2019). Moreover, a cross-sectional study by Luyckx et al. (2013) revealed that the functionality of identity exploration changed from adolescence into emerging adulthood. In adolescence, identity exploration positively predicted commitment making. Yet, in emerging adulthood identity exploration became increasingly associated with depressive symptoms. The current study also supported a positive long-term effect of exploration and openness to identity change in adolescence as a predictor of strong identity commitment in emerging adulthood. Yet, future work is needed to examine whether and how daily identity formation processes change in emerging adulthood and how they relate to adjustment.

### Strengths, Limitations, and Future Directions

This study is characterized by several strengths. First, its longitudinal design included both short-term daily assessments and (bi-) annual long-term assessments of identity, which allowed us to test some key principles of identity theory. Second, this study examined within-person daily identity dynamics. Given the idiosyncratic nature of identity development, it is crucial to study identity at the level were development takes place. Third, this study tested how individual differences in short-term within-person associations were related to individual differences in long-term development. In doing so, this was the first study linking daily identity dynamics in adolescence to identity statuses in emerging adulthood, covering a period of 11 years.

Some limitations warrant attention. First, the present study took a quantitative identity status approach to study identity development. However, a mixed method approach is strongly recommended by including a narrative identity approach as well (McLean et al. 2016). Combining narrative and status approaches could further improve researchers’ understanding on how salient the formation of certain commitments is for individuals. Also, combining the two approaches could inform us whether having established an elaborate identity story facilitates the processes of further strengthening and maintaining these identity commitments on a day-to-day basis (McLean et al. 2016). Second, identity development does not develop in a social vacuum. Yet, the present study did not investigate how crucial contextual variables like the quality of relationships within the family impact daily identity development (e.g., Crocetti et al. 2017). Third, unfortunately, at present, the multi-level time series analytical approach did not allow us to test to what extent the strength of the daily cross-lagged effects changed during adolescence. Theoretically, early adolescents might show a less strong negative dynamic between commitment and reconsideration across days compared to late adolescents. That is, because adolescents might learn that when they have made a certain commitment this implies that other identity choices become less available. Therefore, future work should investigate time (in) variance of daily dynamic effects. Fourth, the majority of participants of this sample came from medium to high SES families. As a result, findings may not generalize to adolescents and emerging adults from more economic disadvantaged families (Phillips and Pittman 2003). Therefore, future studies are needed that include a more diverse sample in terms of SES background.

## Conclusion

Research on identity formation in adolescence typically focused on yearly developmental trajectories. Yet, the short-term daily identity dynamics in adolescence and associations with long-term outcomes of identity development in emerging adulthood have not been explored. The current study addressed these limitations by investigating short-term within person daily dynamics between identity exploration and commitment processes and tested how these processes predicted identity development in emerging adulthood. Results revealed that identity processes to *form* new commitments through exploration as well as processes to *maintain* strong commitments take place within persons across days. By also measuring long-term development of identity in emerging adulthood, this study could assess what type of daily identity dynamics could predict the development of strong and stable identity commitment in emerging adulthood. This study highlights that individual differences in how adolescents deal with identity issues are relatively stable processes across adolescence into emerging adulthood. Of importance, those adolescents who were more flexible in their daily identity formation processes (evidenced by less strong negative daily associations between exploration and commitment making), developed the strongest identity in emerging adulthood. Overall, findings are consistent with the view of continuity in identity development from adolescence into emerging adulthood.

## Supplementary information

Electronic Supplementary Materials
