# Supplementary material for: Daily Identity Dynamics in Adolescence Shaping Identity in Emerging Adulthood: An 11-Year Longitudinal Study on Continuity in Development
Source: J Youth Adolesc. 2021 Jan 9;50(8):1616–33. doi: 10.1007/s10964-020-01370-3 (PMC8270859; doi:10.1007/s10964-020-01370-3)
Supplement: Supplementary file 1 — Electronic Supplementary Materials [file 10964_2020_1370_MOESM1_ESM.docx]

Table S1
*Fit statistics for 1-4 class latent class growth curve models (LCGAs)*

|  |  | **Interpersonal identity** | |  |
| --- | --- | --- | --- | --- |
| fit statistics | 1-class | 2-class | 3-class | 4-class |
| AIC | 7628.891 | 7303.034 | 7158.219 | 7025.443 |
| BIC | 7702.039 | 7417.129 | 7312.643 | 7220.505 |
| ssaBIC | 7644.918 | 7328.273 | 7192.053 | 7068.181 |
| Entropy | - | .802 | .815 | .840 |
| BLRT | - | 0.000 | 0.000 | 0.000 |
| Log likelihood | -3796.446 | -3623.671 | -3541.110 | -3464.722 |
|  |  |  |  |  |
|  |  | **Educational identity** | |  |
| fit statistics | 1-class | 2-class | 3-class | 4-class |
|  |  |  |  |  |
| AIC | 8491.970 | 8156.079 | 8018.336 | 7966.876 |
| BIC | 8573.867 | 8276.975 | 8178.232 | 8165.771 |
| ssaBIC | 8507.243 | 8178.625 | 8048.156 | 8003.969 |
| Entropy | - | .74 | .72 | .76 |
| BLRT | - | 0.000 | 0.000 | 0,000 |
| Log likelihood | -4224.985 | -4047.039 | -3968.168 | -3934.081 |

*Note.* AIC =akaike information criterion; BIC=bayesian information criterion;
ssaBIC=sample size adjusted Bayesian Information Criterion; BLRT=bootstrapped
likelihood ration test.

Table S2

| *Parameter Estimates of Intercept and Slope Factors of Latent Classes for Interpersonal Identity and Educational Identity* | | | | | | | | | | | | | | | | | | | | | | | | | | | | | | | | | |  |
| --- | --- | --- | --- | --- | --- | --- | --- | --- | --- | --- | --- | --- | --- | --- | --- | --- | --- | --- | --- | --- | --- | --- | --- | --- | --- | --- | --- | --- | --- | --- | --- | --- | --- | --- |
|  |  |  |  | | | | | Interpersonal Identity | | | | | |  | |  |  |  |  | | Educational Identity | | | | | | | | | | | | | |
|  |  |  | Identity Moratorium (16%) | |  |  | Identity Achievement  (11%) | | |  |  | Identity Closure  (73%) | |  | |  | Identity Moratorium  (15%) | | | | | | |  |  | Identity Achievement (39%) | | | |  |  | Identity  Closure  (46%) | | |
|  |  |  | *M* | *SE* |  |  | *M* | | *SE* |  |  | *M* | *SE* |  | |  | *M* | | | *SE* | | | |  |  | *M* | *SE* | | |  |  | *M* | *SE* | |
| *Commitment* | |  |  |  |  |  |  | |  |  |  |  |  |  |  |  | | | |  | |  |  |  | | |  |  |  | |  | |  | |
| Mean Int. | |  | 2.37*** | 0.17 |  |  | 4.06*** _a_ | | 0.16 |  |  | 3.65*** _a_ | 0.08 |  |  | 2.73*** | | | | 0.21 | |  |  | 4.07*** | | | 0.16 |  |  | | 3.62*** | | 0.07 | |
| Mean Ls. | |  | 0.28* _ab_ | 0.14 |  |  | 0.29* _a_ | | 0.14 |  | - | 0.01_b_ | 0.04 |  | - | 0.34* | | | | 0.17 | |  |  | 0.10_a_ | | | 0.07 |  |  | | 0.07 _a_ | | 0.08 | |
| Mean Qs. | | - | 0.04 _a b_ | 0.02 |  | - | 0.04 _a_ | | 0.02 |  |  | 0.01 _b_ | 0.01 |  |  | 0.09* | | | | 0.04 | |  | - | 0.01 _a_ | | | 0.01 |  | - | | 0.02 _a_ | | 0.01 | |
| *In-Depth*  *Exploration* | |  |  |  |  |  |  | |  |  |  |  |  |  |  |  | | | |  | |  |  |  | | |  |  |  | |  | |  | |
| Mean Int. | |  | 2.37*** | 0.14 |  |  | 3.71*** _a_ | | 0.20 |  |  | 3.27*** _a_ | 0.08 |  |  | 3.01*** _a_ | | | | 0.13 | |  |  | 3.57*** | | | 0.13 |  |  | | 3.07*** _a_ | | 0.06 | |
| Mean Ls. | |  | 0.14 _a_ | 0.10 |  |  | 0.07 _a_ | | 0.13 |  | - | 0.05 _a_ | 0.04 |  |  | 0.14 _a_ | | | | 0.12 | |  |  | 0.17 _a_ | | | 0.07 |  |  | | 0.13 _a_ | | 0.07 | |
| Mean Qs. | | - | 0.02 _a_ | 0.02 |  | - | 0.00 _a_ | | 0.02 |  |  | 0.01 _a_ | 0.01 |  | - | 0.02 _a_ | | | | 0.03 | |  | - | 0.02 _a_ | | | 0.01 |  | - | | 0.03* _a_ | | 0.01 | |
| *Reconsideration* | |  |  |  |  |  |  | |  |  |  |  |  |  |  |  | | | |  | |  |  |  | | |  |  |  | |  | |  | |
| Mean Int. | |  | 2.23*** _a_ | 0.19 |  |  | 1.51*** | | 0.15 |  |  | 1.93*** _a_ | 0.07 |  |  | 2.97*** | | | | 0.25 | |  |  | 1.76***_a_ | | | 0.13 |  |  | | 2.04*** _a_ | | 0.08 | |
| Mean Ls. | | - | 0.24 _a_ | 0.15 |  | - | 0.16* _a_ | | 0.07 |  | - | 0.14** _a_ | 0.05 |  |  | 0.31 _a_ | | | | 0.23 | |  | - | 0.14 _a_ | | | 0.11 |  | - | | 0.15 _a_ | | 0.10 | |
| Mean Qs. | | - | 0.03 _a_ | 0.03 |  |  | 0.02 _a_ | | 0.01 |  |  | 0.02 _a_ | 0.01 |  | - | 0.11* | | | | 0.04 | |  |  | 0.02 _a_ | | | 0.02 |  |  | | 0.04* _a_ | | 0.02 | |

*Note*. Mean Int. = Mean intercept, Mean Ls. = Mean linear slope, Mean Qs. = Mean quadratic slope.

Means with the same subscript do not differ significantly from one another. Thus, means without a subscript also differ significantly from one another.
******p* <.05, *******p* <.01, ********p* <.001.

Figure S1


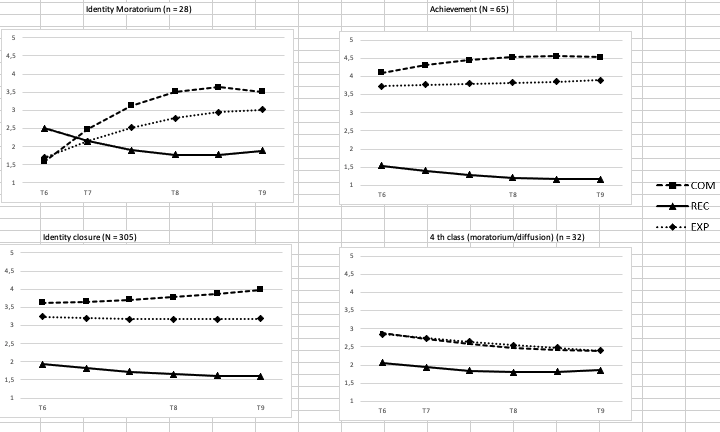


*Figure S1.* 4-class solution for interpersonal identity domain.

Although the fit indices in Table S1 support a 4-class solution compared to a 3-class solution, this class solution not only showed substantial overlap with the 3-class solutions but also included classes that were too small to be used for meaningful replication and subsequent analyses. Specifically, the 4-class solution of the interpersonal identity domain included a class with only 28 participants (7% of the sample). Similarly, the 4-class solution for educational identity included a class with n = 32 participants (9% of the sample). Moreover, Figure S1 and Figure S2 also illustrate the substantial overlap between the fourth class compared to the 3-class solutions. Specifically, Figure S1 shows that the fourth class shows similar levels and development of reconsideration compared to the identity closure status. Besides, this class is somewhere in between the identity moratorium and closures status with regard to commitment and in-depth exploration levels.

Figure S2


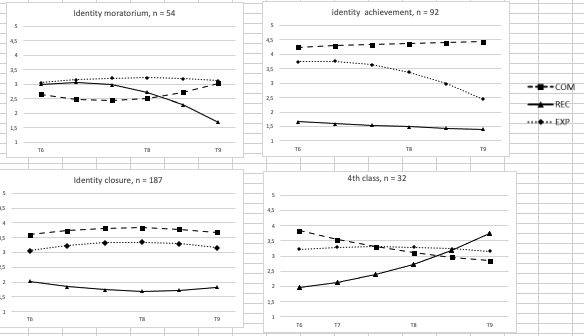


*Figure S2.* 4-class solution for educational identity domain.

Concerning educational identity, the fourth class shows similar initial levels on all identity dimensions compared to the closure status. By adding the fourth class, it seems that the initial identity achievement status is now split into two smaller classes that both maintain relatively high commitments and in-depth exploration, and low reconsideration. Although the slopes of commitment, reconsideration and in-depth exploration are not significantly different from zero in the 4^th^ class the developmental shape suggest increasing identity reconsideration and decreasing commitments over time.

Figure S3

*Figure S2.* Analytical within-person dynamic structural equation model that was used to investigate within-person linkages between daily identity formation processes. COM = daily commitment level, REC = daily reconsideration level, EXP In-Depth = daily exploration in-depth.
